# A Case of Monoclonal Lymphoplasmacytosis of the Bone Marrow with IgM-Positive Russell Bodies

**DOI:** 10.1155/2011/814372

**Published:** 2011-03-07

**Authors:** Hidekazu Kayano, Tsuneyuki Shimada, Naoki Wakimoto, Yuichi Nakamura, Masami Bessho, Hiroshi Yamaguchi, Atsushi Sasaki, Michio Shimizu

**Affiliations:** ^1^Department of Pathology, Faculty of Medicine, Saitama Medical University, 38 Moro Hongoh, Moroyama, Iruma-gun, Saitama 350-0495, Japan; ^2^Department of Hematology, Faculty of Medicine, Saitama Medical University, 38 Moro Hongoh, Moroyama, Iruma-gun, Saitama 350-0495, Japan; ^3^Department of Surgical Pathology, Saitama International Medical Center, 1397-1 Yamane, Hidaka, Saitama 350-1298, Japan

## Abstract

A 71-year-old Japanese male patient infected with HCV was diagnosed with thrombocytopenia. Histological examination of the bone marrow aspirate showed numerous lymphoid aggregates with Russell bodies. Immunohistochemistry and flow cytometric analysis demonstrated clonal expansion of CD5+ CD23+ B cells. Russell bodies were positive for IgM and lambda immunoglobulin light chain. The patient also underwent gastric biopsy, which revealed *Helicobacter pylori* (HP) infection. Subsequent eradication of the bacteria resulted in improvement of his thrombocytopenia. The clinical course remained uneventful at 15-month follow-up, consistent with monoclonal B-cell lymphocytosis. The observed clonal expansion with plasmacytic differentiation may have occurred under the influence of HCV with HP infection.

## 1. Introduction

During histological examination of the bone marrow, lymphoid aggregates are occasionally found. Those lymphoid aggregates may be either neoplastic or reactive, yet the histological distinction can be difficult [1], as it often requires ancillary molecular techniques to detect the clonal population of B-cells in the bone marrow. Although malignant B-cell populations harbor clonal rearrangements of immunoglobulin (Ig) heavy chain genes, clonality of Ig genes is not always sufficient for the diagnosis of malignancy, because some clinically benign diseases, including monoclonal gammopathy of undetermined significance (MGUS) [2] and monoclonal B-cell lymphocytosis (MBL) [3], contain clonal expansion of B-cells. MGUS represents monoclonal expansion of plasma cells with a very low level of abnormal serum protein, whereas MBL is characterized by clonal expansion of B-cells with phenotypes resembling B-cell chronic lymphocytic leukemia (B-CLL) and a very low chance to progress to overt B-CLL.

Some infective agents, such as hepatitis C virus (HCV) and *Helicobacter pylori* (HP), are well known to drive B-cell proliferation, causing different monoclonal or polyclonal lymphoplasmacytic diseases, and this is reviewed by Chiba and Marusawa [4]. In patients infected with HCV, B-cell aggregates are occasionally observed in the bone marrow [1, 5]. Recently, it has been reported that chronic gastritis infected with HP may show accumulation of plasma cells containing Russell bodies (RBs) in the gastric mucosa known as Russell body gastritis [6]. RBs are spherical inclusion bodies in plasma cells occupying the cytoplasm and compressing the nucleus and are thought to be associated with an imbalance between the production and secretion steps of Ig and deposit within vesicular structures derived from dilated rough endoplasmic reticulum [7]. Accordingly, RBs may reflect defects in B-cell development. 

Here, we describe a clonal B-cell population with a CLL-like phenotype aggregated in the bone marrow and show plasmacytic differentiation without clinical progression in a case infected with both HCV and HP.

## 2. Case Report

A 71-year-old Japanese man with chronic hepatitis C infection was referred to our hospital and diagnosed with thrombocytopenia. On admission, physical examination revealed no particular change, such as lymphadenopathy or organomegaly. Laboratory data included white blood cell count, 4, 150 × 10^6^/L; absolute number of lymphocytes, 1, 170 × 10^6^/L; red blood cell count, 495 × 10^10^/L; platelet count, 35, 000 × 10^6^/L; aspartate aminotransferase, 23 IU/L; alanine aminotransferase, 11 IU/L. Total protein was 7.3 g/dL. Antigen of hepatitis B virus was negative, and HCV antibody was positive. No abnormality of serum or urine protein was noted. The differentiated nuclear cells from bone marrow aspirate showed 4% myeloid precursors, 1% myelocytes, 7% metamyelocytes, 34% neutrophils, 3% eosinophils, 0.3% monocytes, 17% lymphocytes, 0.2% plasma cells, and 33% erythroblasts. Although clot section from the aspirated bone marrow showed numerous lymphoid aggregates suggesting lymphomatous involvement, the patient was observed at our clinic without therapy for 15 months. Meanwhile, he also underwent gastric biopsy, which revealed chronic gastritis with HP, but no RB (Figure 3). Subsequent eradication of HP was successful, and his platelet count improved to the level of 130,000 × 10^6^/L. Fifteen months later, bone marrow was aspirated again, revealing the same findings as in the initial examination. His clinical course has been uneventful 22 months after his administration for thrombocytopenia. Bone marrow trephine biopsy was not performed.

## 3. Pathological Findings

**Figure 1:**
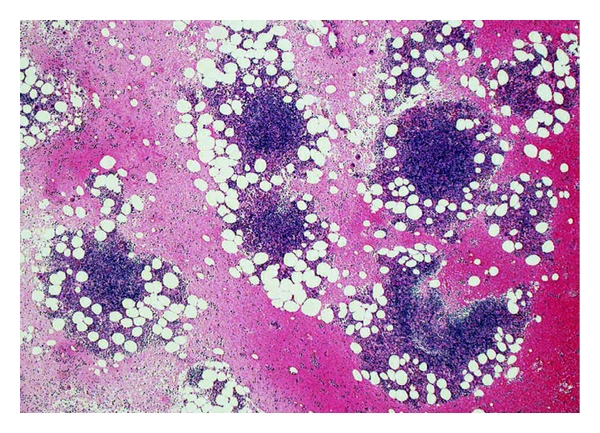
Low-power view of a clot section of bone marrow aspirate (H&E stain). Several lymphoid aggregates are scattered.

**Figure 2:**
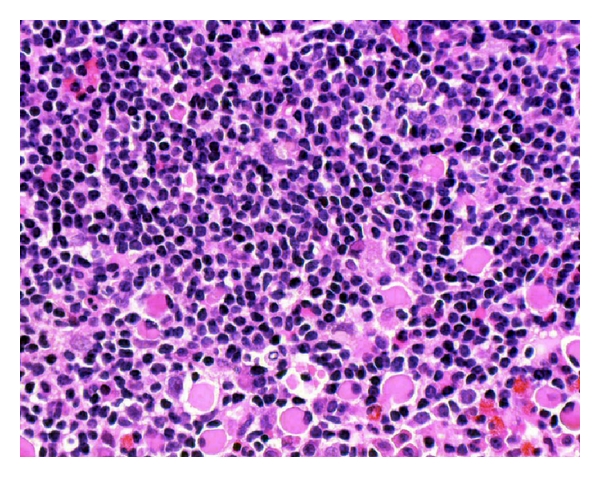
High-power view of lymphoid aggregates in the bone marrow (H&E stain). At the periphery, plasma cells containing Russell bodies are observed.

**Figure 3:**
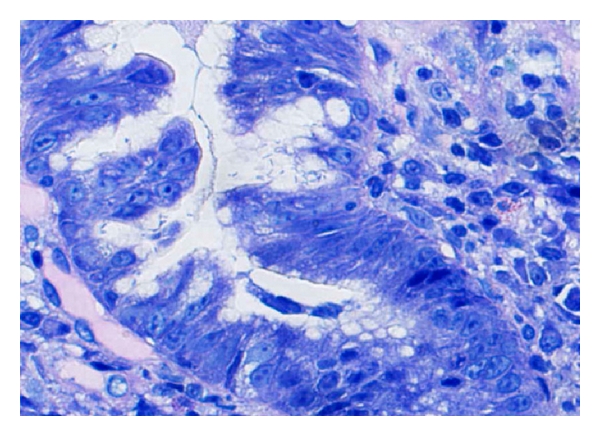
Gastric biopsy showing numerous microorganisms on the foveolar epithelium (Wright-Giemsa stain).

**Figure 4:**
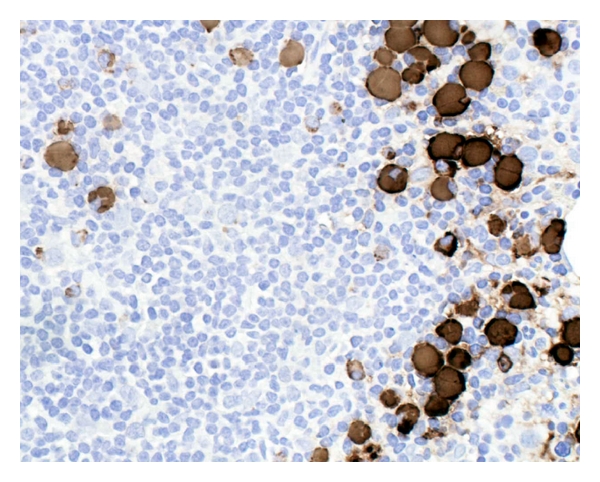
Immunostain for IgM demonstrates intense expression of Russell bodies and some plasma cells.

Clot section from the aspirated bone marrow showed numerous lymphoid aggregates composed of small lymphocytes with numerous Russell bodies at the periphery (Figures 1 and 2) in the background of normocellular marrow with trilineage hematopoiesis and mild erythroid preponderance. Immunohistochemically, the aggregates were composed of CD20+/BCL2+/lambda Ig light chain+ small B-cells with scattered CD3+T-cells. BCL6-positive large B-cells (centroblasts) and CD21-positive follicular dendritic cells revealed germinal centre remnants. At the periphery of the aggregates, almost all of the plasma cells contained Russell bodies positive for IgM (Figure 4) and the lambda Ig light chain, whereas IgD, IgG, IgA, and the kappa light chain were negative. The plasma cells also expressed CD138 and MUM1 but not CD45, CD20, and CD56. Flow cytometric analysis with two-color immunofluorescence on CD45-targeted cell population demonstrated monoclonal B-cells with a CLL-like phenotype, namely, CD5 (76%), CD23 (43%), CD20 (71%), CD19 (74%), CD10 (10%), and monotypic restriction of the lambda Ig light chain (72%). Conventional cytogenetic analysis showed no clonal aberration. Genomic DNA extracted from the paraffin-embedded aspirate was subjected to a seminested polymerase chain reaction (PCR) assay, revealing clonality of the Ig heavy chain gene rearrangement (data not shown). Paraffin-embedded bone marrow aspirate was also subjected to fluorescence in situ hybridization (FISH) analysis using two color fusion probes for *t*(11;14) (Vysis), which revealed no fusion signal of cyclin D1 and the Ig heavy chain genes.

## 4. Discussion

In the present case, clonal expansion of B-cells with a CLL-like phenotype remained in the bone marrow for 15 months without clinical manifestation, consistent with MBL. Of note, each lymphoid aggregate was surrounded by numerous IgM-positive RB, indicating plasmacytic differentiation. Although histological differential diagnosis of the present case would include lymphoplasmacytic lymphoma, characterized by admixture of small lymphocytes and plasma cells with IgM expression, CD5 expression and clinical indolence seen in the present case are not typical of lymphoplasmacytic lymphoma. Mantle cell lymphoma, another CD5-positive B-cell tumor, can be also negated due to the lack of a cytogenetic hallmark or chromosomal translocation involving the CyclinD1 gene.

In western countries, more than 4% of the general population over the age of 40 harbor clonal B-cell populations with an absolute number of peripheral B-cells of less than 5, 000 × 10^6^/L, and the majority of such clonal populations show a CLL (CD5+/CD23+)-like immunophenotype, yet they will not progress to overt CLL. Such a condition has been recognized as MBL, for which the diagnostic criterion is simply based on the detection of a clonal expansion of peripheral B-cells. However, it has been suggested that almost all MBL have some degree of bone marrow involvement, yet detailed understanding of the bone marrow in MBL remains to be clarified [3].

In the pathway of B-cell differentiation, activation-induced cytidine deaminase (AID) is a physiologic genome mutator essential for somatic hypermutation and class switch recombination of Ig genes. AID is induced by nuclear factor kappa B activation via both HCV and HP, causing gene mutations in Ig and non-Ig genes, consistent with the notion that HCV and HP drive B-cell proliferation and directly modulate cellular function, thus giving cell growth advantages and resistance to apoptosis [4]. In fact, HCV infection is associated with proliferation of B-cells expressing CD5 and/or IgM [8], and lymphoid aggregates or less frequent plasmacytosis in the bone marrow is found in over 30% of HCV-infected patients [5]. Thus, it is possible that in our case, CD5-positive B-cells had differentiated to IgM plasma cells under the influence of HCV infection. 

HP infection is also associated with B-cell proliferation such as some gastrointestinal lymphomas. Recently, RB gastritis, a variant of chronic gastritis with numerous RB, has been reported to be associated with HP infection, and the disappearance of RB upon follow-up biopsy after the eradication of HP has been described, thus supporting the causative role of HP in the development of RB [6]. In the present case, however, the impact of HP infection on the RB formation is yet to be known since the bone marrow aspirate repeated after eradication of HP revealed RB as in the initial examination.
